# The Prophylaxis Effect of Ephedrine on Hemodynamic Variation in Patients Undergoing Percutaneous Nephrolithotomy Surgery with Spinal Anesthesia

**DOI:** 10.1155/2023/8966501

**Published:** 2023-02-24

**Authors:** Shahryar Sane, Ali Akbar Nasiri, Ayatay Bahrami, Zaynitdin Kamalov, Shadia Hamoud Alshahrani, Hadi Sajid Abdulabbas, Shahram Darvishzadehdaledari, Parang Golabi, Behzad Kazemi Haki

**Affiliations:** ^1^Department of Anesthesiology, Urmia Imam Khomeini Hospital, Urmia University of Medical Science, Urmia, Iran; ^2^Department of Medicine, Urmia Imam Khomeini Hospital, Urmia University of Medical Science, Urmia, Iran; ^3^Department of Immunoregulation, Institute of Immunology and Human Genomics, Academy of Science of the Republic of Uzbekistan, Yakhyo Gulamov Str. 74, Tashkent, Uzbekistan; ^4^Medical Surgical Nursing Department, King Khalid University, Almahala, Khamis Mushate, Saudi Arabia; ^5^Continuous Education Department, Faculty of Dentistry, University of Al-Ameed, Karbala 56001, Iraq; ^6^Department of Health Sciences, University of York, Heslington, York, UK; ^7^Department of Anesthesiology, Omid Charity Hospital, Urmia University of Medical Science, Urmia, Iran

## Abstract

**Background:**

Performing spinal anesthesia with at least hemodynamic variation and complications is always challenging for anesthesiologists. In this study, we investigated the effect of ephedrine and placebo on hemodynamic changes in patients undergoing percutaneous nephrolithotomy with spinal anesthesia.

**Methods:**

This randomized, double-blind prospective clinical trial was conducted on 120 patients aged 20‒60 years with ASA (American Society of Anesthesiologists) classes I and II. Patients who were candidates for percutaneous nephrolithotomy with spinal anesthesia were divided into intervention (received 1 cc = 5 mg ephedrine) and control groups (received 1 cc normal saline). All vital parameters, including HR (heart rate) and NIBP (noninvasive blood pressure), were recorded perioperatively T0–T25) and finally at the end of surgery time (Tf). The results were analyzed by SPSS software version 23, and a *P* value ≤0.05 was considered significant.

**Results:**

The mean arterial pressure during surgery between T3 and T9 and the mean heart rate in times of T3–T8 in the intervention group were higher than in the control group, and this difference was statistically significant (*P* < 0.05). The incidence of hypotension, bradycardia, nausea, and vomiting and the amount of prescribed ephedrine, atropine, and ondansetron in the control group were higher than in the intervention group (*P*=0.001). Seven patients in the control group and four in the intervention group had shivering, but this difference was not statistically significant (*P*=0.43).

**Conclusion:**

This study showed the effectiveness of the prescription of 5 mg ephedrine two minutes before changing from the lithotomy position to the supine in maintaining hemodynamic stability, reducing hypotension, bradycardia, nausea, and vomiting, and the amount of prescribed ephedrine, atropine, and ondansetron. *Trial Registrations*. This trial is registered with IRCT20160430027677N22.

## 1. Introduction

The core issue that anesthesiologists consider in all operations is patient safety and hemodynamic stability in anesthesia [1–3]. Regional anesthesia is the preferred method of anesthesia for lower abdominal, lower limb, urology, gynecology, and anorectal surgery [4]. This method's advantages include reducing patient mortality and complications, better analgesia during and after the operation, and economic superiority over general anesthesia [5]. Percutaneous nephrolithotomy (PCNL) is a minimally invasive surgical procedure applied to kidney stone removal. It is an effective and safe method for kidney stone removal with different structures. It was first proposed in urology in 1976 and replaced open surgical treatment for removing stones in the upper part of the urinary tract [6]. Because percutaneous stone surgery has less morbidity, a shorter recovery, and less cost, it is currently considered the preferred treatment for most stones [7]. It seems that in recent years, facing severe complications, urologists are attempting to develop further and improve the percutaneous nephrolithotomy (PCNL) method to use it as a safe and low-complication method for treating kidney stones [6, 7].

During this surgery, patients are placed in two different positions. First, the patient is placed in the lithotomy position (after induction of general anesthesia or spinal anesthesia), and in the continuation of the surgery, they are placed in the prone or lateral position to perform percutaneous nephrolithotomy (PCNL). Both positions will bring about hemodynamic changes for patients, and patients with coexistent diseases such as cardiovascular disease or diabetes can cause severe complications [8]. Today, the preferred anesthesia method in these surgeries is intrathecal anesthesia, according to the observed results, compared to general anesthesia [5, 8]. Ephedrine is a noncatecholamine sympathomimetic amine drug that is used intravenously. Ephedrine increases blood pressure and HR (heart rate). It is also used orally as a bronchodilator due to the direct stimulating effects of beta2-adrenergic receptors. Its dose is 2.5–25 mg intravenously or 25–50 mg intramuscularly [9, 10].

Some studies illustrated that using 0.5–0.75 *μ*g/kg dexmedetomidine reduced the incidence and severity of bradycardia in percutaneous nephrolithotomy [11]. Roodneshin et al.' [12] study showed that choosing the optimal position in the PCNL technique depends on the patient's condition. If hemodynamic control matters to the anesthesiologist, then lateral positions are more appropriate. However, if pain control and a long time of analgesia are essential, a prone position may be preferred. Prophylactic ephedrine versus prophylactic ondansetron was compared in percutaneous nephrolithotomy (PCNL) by Nada and Hegab. During percutaneous nephrolithotomy (PCNL) operation, ondansetron 6 mg administered 5 minutes before spinal anesthesia was comparable to 15 mg ephedrine administered immediately after block in attenuating hypotension and bradycardia, as well as declined the number of patients who required rescue vasoconstrictors during surgery [13]. A clinical trial in Egypt assessed the effect of ondansetron on spinal-induced hypotension during PCNL operation. According to the study, ondansetron 4 mg IV (intravenous) 5 minutes before spinal anesthesia had no impact on reducing the incidence of hypotension, and also, the amount of ephedrine needed during PCNL under spinal anesthesia was not declined [14].

Considering the different results obtained regarding the effect of ephedrine on hemodynamic changes during various surgeries in clinical studies and also the recommendation of the researchers in the same studies to perform more studies in this field, in this study, we investigated the effect of ephedrine and placebo on hemodynamic changes in patients undergoing percutaneous (PCNL) nephrolithotomy with spinal anesthesia.

## 2. Methods

### 2.1. Study Design

**Figure 1:**
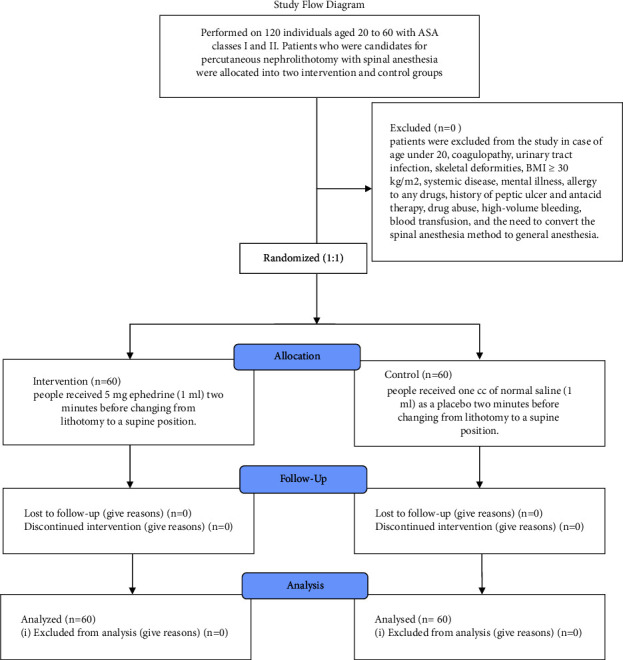
Study flow diagram.

This randomized, double-blind prospective clinical trial study was approved by the Research and Ethics Committee of the Urmia University of Medical Sciences (IR.UMSU.REC.1401.182) and registered in the Iranian Registry of Clinical Trials (IRCTID: IRCT20160430027677N22). This study was conducted on 120 patients aged 20‒60 years with ASA (American Society of Anesthesiologists) classes I and II. Patients who were candidates for percutaneous (PCNL) nephrolithotomy with spinal anesthesia were divided into two intervention and control groups (Figure 1). According to the random number table, patients were randomly assigned to two groups, and the anesthesiologist and staff were unaware of which patient belonged to which group.

### 2.2. Subjects and Setting

All patients were visited by an anesthesiologist the day before the surgery. Patients were kept fasting for at least 8 hours before surgery. Syringes were identical in appearance, and all of them were encoded, and the surgeon, staff, and anesthesiologist were unaware of the contents of the syringes. A standard pulse oximetry monitor, a noninvasive blood pressure (NIBP) measurement device, and an ECG (electrocardiogram) were connected in the operating room. After an 18 cm venous catheter was inserted, 10 ml/kg of ringer lactate was infused. Baseline heart rate (HR), blood pressure, and oxygen saturation were recorded.

### 2.3. Inclusion Criteria

Patients who were candidates for PCNL elective surgery with pilocalyx stones larger than 20 mm and 10–20 mm stones resistant to ESWL (extracorporeal shock wave lithotripsy), lower calyx stones larger than 10 mm, large impacted proximal ureter stones, body mass index <30 kg/m^2^·h, aged 20–60, American Society of Anesthesiologists (ASA) classes I and II, and patients who have signed the consent form to participate in the trial were included.

### 2.4. Exclusion Criteria

Patients aged under 20 years old, coagulopathy, untreated active urinary tract infection, patients with skeletal deformities, body mass index ≥30 kg/m^2^, history of systemic disease, mental illness, history of allergy to any of the drugs used during the study, history of peptic ulcer disease and antacid therapy, and drug abuse were excluded. Also, patients were excluded from the study in case of high-volume bleeding, blood transfusion, and the need to convert the spinal anesthesia method to general anesthesia.

### 2.5. Intervention Design

#### 2.5.1. Intervention Group

60 people received 5 mg ephedrine (1 ml) two minutes before changing from lithotomy to a supine position.

#### 2.5.2. Control Group

60 people received one cc of normal saline (1 ml) as a placebo two minutes before changing from lithotomy to supine position.

Each ephedrine syringe contained 50 mg of ephedrine diluted with 9 ml of distilled water in 10 cc syringes, each cc containing 5 mg of ephedrine. The placebo syringes were in the form of 10 cc syringes containing distilled water, and two minutes before changing the lithotomy position to the supine position, one cc of each was randomly injected intravenously into the patients in the study groups. The patients, surgeons, and anesthesiologists were blinded to the allocation of the patients to the studied groups. All aseptic precautions were conducted before performing the spinal anesthesia. Spinal anesthesia was performed with a #25 needle (SPINAL ANESTHESIA NEEDLE, Dr.J brand, made in Japan, Quincke type) in the sitting position from the third and fourth intervertebral space (midline approach) and by injecting 12.5 mg hyperbaric bupivacaine in the subarachnoid space. Then, the patients were placed in the supine position and received 4 liters of oxygen per minute during the operation through a simple face oxygen mask. Sensory levels were determined by the pinprick test after block (every 15–20 seconds for 3 minutes), and the motor blockade was evaluated using Bromage's criteria until the level of spinal anesthesia was raised to the T8 level.

Cystoscopy was performed in lithotomy position, a ureteric catheter was entered into the upper ureter or renal pelvis and fixed with tape to the indwelling Foley catheter, and patients were placed in the supine position. The patient's supine position changed to the prone position, and renal access was conducted in the prone position under fluoroscopic guidance; superior and inferior bolsters were placed at the xiphoid process cartilage to support the lower rib cage, and at the symphysis pubis, vertical bolsters were put in the standard manner along the lateral sides of the chest.

All vital parameters, including HR and NIBP, were recorded perioperatively the time before spinal anesthesia performing (T0), immediately after spinal anesthesia induction (T1), after the lithotomy position (T2), when lithotomy position changed to the supine position (T3), and then when the patient was placed in the prone position (T4). Afterward, vital signs were documented every 3 minutes for 60 minutes (T5–T25) and finally at the end of surgery time (Tf).

If the systolic blood pressure was under 100 mmHg or less than 20% from the baseline, it was treated with 5 mg ephedrine and increased crystalloid speed. If the heart rate (HR) was under 50 beats/minute, it was treated with 0.75 mg of atropine. The incidence of hypotension, bradycardia, nausea, vomiting, shivering, and other complications were recorded.

### 2.6. Primary Outcomes

The blood pressure and heart rate (HR) variation between the two groups were measured and recorded in the checklist as the primary outcome.

### 2.7. Secondary Outcomes

The secondary outcome was the amount of prescribed ephedrine, nausea, and vomiting intraoperatively and postoperatively, which was recorded in a checklist.

### 2.8. Statistical Analysis

Based on the Nada and Hegab' study in 2018 [13], considering the power (probability) test of 80%, confidence interval of 95% (*α* = 0.05 *β* = 20%), and a dropout risk of 20%, we enrolled 60 patients in each group. Tables, frequency charts, and descriptive statistics, including mean and standard deviation, were used to provide descriptive features. The repeated measures test was used for normal data to compare the mean arterial pressure and heart rate at T0–T25 and Tf. Furthermore, the Friedman test was used for non-normal data. This study used the chi-square test to investigate qualitative variables such as gender. Moreover, for quantitative variables in two groups, an independent *t*-test was used for normal data. For non-normal data, the Mann–Whitney test was used. The normality of data was tested using the Kolmogorov‒Smirnov test. The results were analyzed by SPSS software version 23, and a *P* value ≤0.05 was considered significant.

## 3. Results

Chi-square and *t*-tests did not show any significant difference between the patients' demographic information regarding age, gender, and stone characteristics. It confirmed the homogeneity of the patients in terms of demographic characteristics in the two groups and the generalizability of the results.

The patients' demographic information in the two groups is presented in Table 1. According to the *t*-test and chi-square test, there was no statistically significant difference between the two groups' characterized data, including gender, BMI (body mass index), age, and location of the stone (*P* > 0.05).

### 3.1. Mean Arterial Pressure Changes

**Figure 2:**
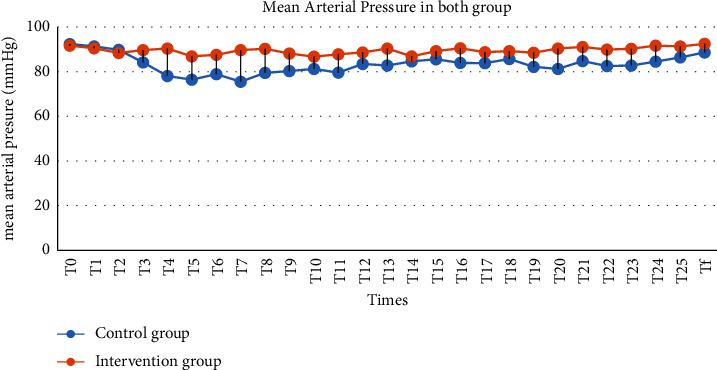
Mean arterial blood pressure variation in both groups.

The mean arterial pressure changes during surgery between T3 and T9 in the intervention group were higher than in the control group, and this difference was statistically significant (*P* < 0.05). Also, at other times during the study (except for T0, T1, and T2), the mean arterial pressure was higher than the control group. However, it was not statistically significant (*P* > 0.05, Table 2 and Figure 2).

### 3.2. Heart Rate Variation

**Figure 3:**
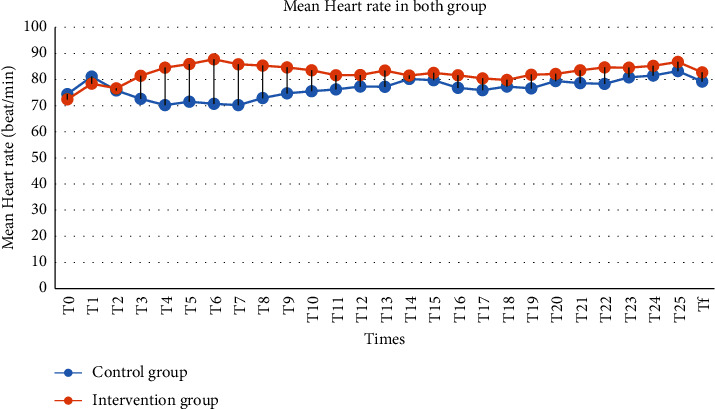
Mean heart rate variation in both groups.

The mean heart rate (HR) variations at the onset of study T0 and T1-T2 were approximately equal in both groups; but in the intervention group, heart rate was higher than that in the control group in times of T3–T8, and this difference was statistically significant (*P* < 0.05). In other times (T10-Tf), which evaluated during the study, the mean heart rate (HR) was higher than the control group. However, in the conducted independent *t*-test, it was not significant (*P* > 0.05, (Table 3 and Figure 3).

The incidence of hypotension, bradycardia, nausea, and vomiting and the amount of prescribed ephedrine, atropine, and ondansetron in the control group were higher than in the intervention group, and the difference was statistically significant (*P*=0.001). There was no blood transfusion required in both groups; in the conducted independent *t*-test, it was not significant (*P*=1.00). Seven patients in the control group and four in the intervention group had shivering, but this difference was not statistically significant (*P*=0.438) (Table 4).

## 4. Discussion

There is no doubt about the low risk of the spinal anesthesia method over general anesthesia [15]. However, performing spinal anesthesia with at least hemodynamic variation and complications is always challenging for anesthesiologists. They always endeavor to crack this problem and provide excellent spinal anesthesia for patients, especially when patients should be placed in prone positions for surgery. PCNL (percutaneous nephrolithotomy) is one of the most common low-intervention surgeries for removing complex and large kidney calculi [16] performed under spinal anesthesia. This operation may be performed in various positions, but the prone position is the most common in percutaneous endourological surgery. In this clinical trial, we investigated the effect of ephedrine (5 mg) in maintaining hemodynamic stability in patients who were candidates for elective PCNL surgery in the prone position.

Our findings demonstrated that the mean arterial pressure (MAP) during surgery between the time of lithotomy position changed to the supine position (T3) and when the patient was placed in the prone position (T4) up until T9 time in the intervention group were significantly higher than that in the control group. However, in the whole study period, except for T0–T2, the mean arterial pressure in the intervention group was higher than that in the control group, but it was not statistically significant. Also, the mean heart rate in the intervention group was higher than that in the control group, but it was statistically significant only in times T3–T8. Kamal et al. [17] compared 1298 PCNL operations, of which 1160 patients underwent PCNL under spinal anesthesia. The results revealed that only in the first 10 minutes of anesthesia, 148 (12.75%) patients developed hypotension, which was managed by ephedrine intravenously (IV); these findings were compatible with our results. Movassaghi et al. [18] investigated spinal and general anesthesia for PCNL. There was no statistical difference between the two groups regarding trends of systolic blood pressure (SBP), diastolic blood pressure (DBP), mean arterial pressure (MAP), and heart rate (HR) (*P*=0.990, *P*=0.568, *P*=0.710, and *P*=0.934, respectively, from repeated measurements). However, in operation time-to-time analysis, SBP was significantly lower in general anesthesia group only in 120th minute, DBP in 60^th^, 90^th^, and 120^th^ minutes, and MAP in 90^th^ and 120^th^ minutes (*P* < 0.05) [18]. Nada and Hegab evaluated the effects of prophylactic ephedrine versus ondansetron on postspinal hemodynamic variation in percutaneous nephrolithotomy (PCNL). Their trial illustrated that although mean arterial pressures (MAP) and heart rate (HR) were lower in the ondansetron group, there was no significant difference between the groups regarding the proportion of patients experiencing hypotension and ephedrine consumption [13]. These findings were not consistent with our results; probably, this was due to the different positions of patients in the two trials. In the Nada and Hegab' study, patients were placed in a free-flank modified supine position, but in our study, patients were placed in the prone position. Roodneshin et al. studied the effect of lateral versus prone position on hemodynamic stability and pain control in patients undergoing percutaneous nephrolithotomy (PCNL). The results showed that the amount of ephedrine usage in the lateral group (3.6 ± 1.5 mg) was significantly lower than that in the prone group (16.4 ± 12 mg), suggesting more hemodynamic variations in the prone group during the operation [12]. However, a clinical trial conducted by Elnagar et al. assessed the effect of ondansetron on spinal-induced hypotension during PCNL; the trial demonstrated that ondansetron 4 mg intravenously 5 minutes before spinal anesthesia had no impact on reducing the incidence of hypotension and the required dose of ephedrine [19].

In our study, the incidence of hypotension, bradycardia, nausea, and vomiting, as well as the amount of prescribed ephedrine, atropine, and ondansetron in the control group, was higher than that in the intervention group. This difference was statistically significant (*P* = 0.001). Mehrabi and Shirazi analyzed the outcomes and complications of spinal anesthesia in percutaneous nephrolithotomy (PCNL) in 160 patients; during the first phase after inducing spinal anesthesia, 18 patients developed hypotension, which was treated with 10 mg ephedrine intravenously. Ten patients required a blood transfusion (6.3%), and six experienced mild to moderate headache, dizziness, and mild low back pain for 2‒4 days after the operation, which was treated with analgesics and bed rest [20]. This study had similar results regarding the incidence of hypotension to our clinical trial, but in our research, no blood transfusion was required in both groups. This was probably due to less stone bulk in our study than in Elnagar et al. [19] study, which was 34.2 ± 9.8 mm. It has been reported that the blood transfusion rate during PCNL is about 3.5%‒9.29% [21, 22]. Jain et al. investigated prophylactic phenylephrine and ephedrine infusion during spinal anesthesia for an emergency cesarean. The study revealed a significant increase in heart rate from the baseline to 9 minutes after induction in the ephedrine group, and maternal bradycardia occurred in the phenylephrine group (*P* = 0.02). On the other hand, heart rate decreased significantly from baseline after the third minute in the phenylephrine group. As well as, the trial indicated that the incidence of maternal nausea and vomiting was higher in the ephedrine group than that in the phenylephrine group (22.2% vs. 4.4%; *P* = 0.02) [23]. In the study of Nada and Hegab [13], the incidence of nausea and vomiting in the ephedrine group was higher than that in the ondansetron group, and this difference was statistically significant (*P* = 0.019). In our investigation, the prescription of 5 mg ephedrine (1 ml) for two minutes before changing from lithotomy to supine position had a reasonable impact on the incidence of nausea and vomiting in the intervention group than that in the control group; and this difference was statistically significant. This difference between our study and with two trials mentioned above [13, 23] is probably due to the kind of surgery; pregnancy affects many organs and viscera, especially the digestive system, such as the stomach, so in cesarean surgery, patients are considered full stomach and they are at the risk of vomiting. It has been proven that the patients' positions during surgery are related to the incidence of nausea and vomiting, whereas the incidence of nausea and vomiting in the supine and prone positions was higher than that in lateral and free-flank positions [24–26].

In our trial, seven patients in the control group and four in the intervention group had shivering. However, this difference was not statistically significant (*P*=0.438). In a study in Egypt [13] that compared the effect of ephedrine and ondansetron on the hemodynamics in the patients undergoing PCNL surgery, the results demonstrated that the ondansetron had an excellent antishivering effect than ephedrine (*P*=0.002). El-Deeb and Barakat [27] attempted to answer whether ephedrine replaces meperidine to prevent shivering in women undergoing cesarean section under spinal anesthesia. Their study illustrated that the incidence of shivering in meperidine and ephedrine groups in women undergoing cesarean section under spinal anesthesia was comparable (27% and 29%, respectively, *P*=0.06). Also, the intensity of shivering was not different between the two groups. Ephedrine is a sympathomimetic agent with an antishivering effect and an antiemetic effect in the short term [27, 28].

## 5. Conclusion

This study showed the effectiveness of the prescription of 5 mg ephedrine two minutes before changing from the lithotomy position to the supine in maintaining hemodynamic stability, reducing hypotension, bradycardia, nausea, and vomiting, and the amount of prescribed ephedrine, atropine, and ondansetron.

### 5.1. Study Limitation

Our investigation restriction was generalizability because this clinical trial was conducted in a small area. Furthermore, due to the different doses of ephedrine used in other clinical trials, determining accurate doses needs more investigations in different geographical regions.

### 5.2. Suggestions

Conducting more research in this field and a combination of clinical knowledge and experience to generalize the results of evidence-based studies are suggested.

## Figures and Tables

**Table 1:** Studied patients' demographic data, size, and location of stones.

	Intervention group		Control group		*P* value
	*N* = 60 patients		*N* = 60 patients		
Gender (F/M)	Female	Male	Female	Male	**0.752**
	34	26	32	28	

Age (year)	36.23 ± 11.31		35.17 ± 12.28		**0.464**

ASA classes I and II	Class I	Class II	Class I	Class II	**0.226**
	24	36	28	32	

Stone side	Left kidney	Right kidney	Left kidney	Right kidney	**0.829**
	27	33	30	30	

Size of kidney stone (mm)	31.15 ± 2.33		29.56 ± 3.12		**0.437**
BMI (kg/m^2^)	26.12 ± 3.32		25.45 ± 3.56		**0.516**
Duration of surgery (min)	81.41 ± 7.11		83.12 ± 5.37		**0.489**

Values are presented as mean ± SD (standard deviation) or number. There were no significant differences between the two groups (*P* > 0.05).

**Table 2:** Mean arterial blood pressure variation in both groups.

Time	Control group	Intervention group	*P* value
T0	92.43 ± 2.24	91.73 ± 3.17	0.316
T1	91.32 ± 3.11	90.55 ± 3.42	0.074
T2	89.76 ± 2.85	88.42 ± 2.14	0.241
T3	84.15 ± 4.45	89.64 ± 4.26	**0.042**
T4	78.13 ± 3.72	90.38 ± 4.56	**0.016**
T5	76.45 ± 5.52	86.88 ± 3.62	**0.001**
T6	78.92 ± 5.76	87.62 ± 5.22	**0.023**
T7	75.53 ± 6.32	89.73 ± 4.83	**0.012**
T8	79.44 ± 4.35	90.23 ± 2.18	**0.034**
T9	80.35 ± 5.79	88.17 ± 3.92	**0.033**
T10	81.26 ± 3.61	86.72 ± 3.87	0.228
T11	79.65 ± 5.37	87.82 ± 2.79	0.364
T12	83.51 ± 4.92	88.62 ± 3.69	0.092
T13	82.77 ± 4.15	90.36 ± 3.81	0.141
T14	84.69 ± 3.47	86.87 ± 2.78	0.417
T15	85.56 ± 4.26	89.24 ± 4.38	0.638
T16	83.92 ± 5.41	90.54 ± 2.59	0.542
T17	83.78 ± 6.29	88.73 ± 3.63	0.098
T18	85.68 ± 4.87	89.24 ± 1.92	0.127
T19	82.15 ± 4.69	88.47 ± 3.77	0.443
T20	81.22 ± 5.44	90.41 ± 2.14	0.093
T21	84.79 ± 3.85	91.13 ± 3.66	0.642
T22	82.49 ± 7.23	89.92 ± 4.73	0.087
T23	82.76 ± 6.78	90.25 ± 4.66	0.519
T24	84.52 ± 5.79	91.65 ± 3.61	0.667
T25	86.36 ± 5.52	91.29 ± 4.36	0.851
Tf	88.61 ± 4.58	92.46 ± 4.28	0.265

Values are presented as mean ± SD (standard deviation) or number.

**Table 3:** Mean heart rate variation in both groups.

Time	Control group	Intervention group	*P* value
T0	74.43 ± 5.69	72.39 ± 6.81	0.213
T1	81.12 ± 4.35	78.42 ± 4.73	0.471
T2	75.82 ± 4.89	76.61 ± 5.38	0.181
T3	72.63 ± 5.76	81.47 ± 3.57	**0.021**
T4	70.22 ± 5.82	84.55 ± 5.79	**0.001**
T5	71.51 ± 6.86	85.92 ± 5.66	**0.001**
T6	70.68 ± 5.29	87.77 ± 4.96	**0.032**
T7	70.19 ± 4.47	85.82 ± 4.18	**0.041**
T8	72.95 ± 4.78	85.37 ± 3.25	**0.025**
T9	74.76 ± 6.52	84.66 ± 4.61	0.273
T10	75.49 ± 4.58	83.58 ± 5.49	0.128
T11	76.18 ± 5.63	81.65 ± 4.12	0.364
T12	77.32 ± 3.74	81.73 ± 3.59	0.251
T13	77.24 ± 3.67	83.48 ± 4.52	0.732
T14	80.25 ± 4.32	81.54 ± 3.97	0.164
T15	79.69 ± 5.83	82.59 ± 5.66	0.521
T16	76.83 ± 7.24	81.62 ± 4.56	0.247
T17	75.88 ± 6.31	80.47 ± 6.15	0.652
T18	77.32 ± 4.75	79.85 ± 6.54	0.284
T19	76.65 ± 4.68	81.87 ± 4.36	0.617
T20	79.41 ± 5.16	82.12 ± 3.26	0.359
T21	78.63 ± 4.11	83.53 ± 5.48	0.242
T22	78.33 ± 5.28	84.68 ± 5.41	0.583
T23	80.79 ± 6.33	84.51 ± 4.37	0.458
T24	81.54 ± 4.52	85.23 ± 3.39	0.475
T25	83.26 ± 4.35	86.74 ± 4.56	0.148
Tf	79.23 ± 4.83	82.78 ± 5.18	0.373

Values are presented as mean ± SD (standard deviation) or number. The time between T3 and T8 (*P* values) is bolded.

**Table 4:** The incidence of study complications in both groups.

Variables	Control group	Intervention group	*P* value
Incidence of hypotension (mean SBP (systolic blood pressure) < 100) (*n*)%	18 (30.00%)	2 (3.33%)	0.001
Incidence of bradycardia (pulse < 50) (*n*)%	8 (13.33%)	0 (0.00%)	0.002
Incidence of nausea or vomiting	16 (26.66%)	3 (5.00%)	0.001
Prescribed vasopressors (5 mg ephedrine) (*n*)	18 (30.00%)	2 (3.33%)	0.011
Prescribed atropine (0.75 mg) (*n*)	8 (13.33%)	0 (0.00%)	0.031
Prescribed ondansetron (*n*)	16 (26.66%)	3 (5.00%)	0.025
Blood loss requiring transfusion	0 (0.00%)	0 (0.00%)	1.00
Shivering	7 (11.66%)	4 (6.66%)	0.438

## Data Availability

All relevant data are included within the article. Additional information is available from the corresponding author upon request.
